# Supplementary material for: Does delaying discharge from intensive care until after tracheostomy removal affect 30-day mortality? Propensity score matched cohort study
Source: BMJ Open. 2020 Jun 7;10(6):e037762. doi: 10.1136/bmjopen-2020-037762 (PMC7282394; doi:10.1136/bmjopen-2020-037762)
Supplement: Supplementary data [file bmjopen-2020-037762supp001.pdf]

SUPPLEMENTARY MATERIAL: Balance in potential confounders

Table S1 Balance in potential confounders before and after matching – cases discharged to a ward

Potential confounder	Before matching			After matching		
	Discharged with tracheostomy (N=70)	Remained in ICU (N=960[a])	Standardised difference	Discharged with tracheostomy (N=70)	Remained in ICU (N=70[b])	Standardised difference
Age (years), mean (SD)	65.0 (13.3)	63.3 (12.9)	13.1	65.0 (13.3)	66.3 (10.3)	10.9
Male, n (%)	50 (71.4)	607 (63.2)	17.5	50 (71.4)	48 (68.6)	6.2
APACHE II APS, mean (SD)	14.7 (6.2)	13.2 (6.5)	25.0	14.7 (6.2)	14.9 (6.7)	3.3
APACHE II Score, mean (SD)	19.5 (7.1)	17.7 (7.0)	25.8	19.5 (7.1)	20.0 (7.4)	6.7
Surgical status – elective/scheduled, n (%)	2 (2.9)	51 (5.3)	12.4	2 (2.9)	2 (2.9)	0
Surgical status – emergency/urgent, n (%)	10 (14.3)	139 (14.5)	0.5	10 (14.3)	10 (14.3)	0
Neurological reason for ventilation, n (%)	11 (15.7)	94 (9.8)	17.7	11 (15.7)	10 (14.3)	4.0
Days since tracheostomy, mean (SD)	15.7 (11.0)	17.2 (13.7)	11.8	15.7 (11.0)	15.5 (11.8)	1.5
Days receiving sedatives, mean (SD)	7.6 (5.1)	8.9 (6.7)	23.1	7.6 (5.1)	7.5 (5.3)	1.6
Days of advanced respiratory support, mean (SD)	14.7 (9.4)	19.1 (14.3)	36.0	14.7 (9.4)	14.6 (12.3)	0.9
Days of basic respiratory support, mean (SD)	6.1 (7.7)	5.6 (9.7)	6.0	6.1 (7.7)	6.3 (11.0)	1.8
Days of circulatory support, mean (SD)	3.2 (3.4)	4.1 (4.6)	23.5	3.2 (3.4)	2.6 (2.7)	20.0
Days of neurological support, mean (SD)	3.7 (6.2)	2.3 (5.1)	26.1	3.7 (6.2)	2.9 (4.3)	16.0
Days of renal support, mean (SD)	0.9 (4.6)	1.6 (5.4)	14.1	0.9 (4.6)	0.7 (2.6)	7.3
Receiving sedatives on final day, n (%)	6 (8.6)	83 (8.6)	0.3	6 (8.6)	4 (5.7)	11.0
Advanced respiratory support on final day, n (%)	4 (5.7)	310 (32.3)	71.9	4 (5.7)	5 (7.1)	5.8
Basic respiratory support on final day, n (%)	46 (65.7)	593 (61.8)	8.2	46 (65.7)	40 (57.1)	17.6
Circulatory support on final day, n (%)	0 (0)	32 (3.3)	26.2	0 (0)	0 (0)	N/A
Neurological support on final day, n (%)	6 (8.6)	11 (1.1)	34.8	6 (8.6)	2 (2.9)	24.6
Renal support on final day, n (%)	0 (0)	8 (0.8)	13.0	0 (0)	0 (0)	N/A

[a]960 days of observation from 292 patients; [b]49 unique patients after matching with replacement

APACHE, Acute Physiology And Chronic Health Evaluation; APS, Acute Physiology Score; SD, standard deviation

Table S2 Balance in potential confounders before and after matching – cases discharged to an HDU

Potential confounder	Before matching			After matching		
	Discharged with tracheostomy (N=74)	Remained in ICU (N=999[a])	Standardised difference	Discharged with tracheostomy (N=74)	Remained in ICU (N=74[b])	Standardised difference
Age (years), mean (SD)	64.1 (11.8)	62.7 (13.1)	11.0	64.1 (11.8)	64.5 (11.9)	3.5
Male, n (%)	32 (43.2)	644 (64.5)	43.4	32 (43.2)	56 (75.7)	69.5
APACHE II APS, mean (SD)	14.1 (5.5)	13.4 (6.5)	11.5	14.1 (5.5)	12.5 (6.5)	26.5
APACHE II Score, mean (SD)	18.9 (6.5)	17.9 (7.0)	15.5	18.9 (6.5)	17.7 (7.0)	18.3
Surgical status – elective/scheduled, n (%)	6 (8.1)	51 (5.1)	12.1	6 (8.1)	8 (10.8)	9.2
Surgical status – emergency/urgent, n (%)	14 (18.9)	140 (14.0)	13.2	14 (18.9)	15 (20.3)	3.4
Neurological reason for ventilation, n (%)	7 (9.5)	100 (10.0)	1.9	7 (9.5)	9 (12.2)	8.7
Days since tracheostomy, mean (SD)	13.6 (9.9)	17.2 (13.6)	30.5	13.6 (9.9)	12.8 (9.8)	7.9
Days receiving sedatives, mean (SD)	8.7 (8.2)	9.1 (6.9)	6.1	8.7 (8.2)	8.2 (6.0)	6.8
Days of advanced respiratory support, mean (SD)	15.9 (12.2)	19.0 (14.1)	23.5	15.9 (12.2)	15.6 (11.5)	2.5
Days of basic respiratory support, mean (SD)	5.8 (11.0)	5.5 (9.5)	2.7	5.8 (11.0)	7.4 (12..8)	13.2
Days of circulatory support, mean (SD)	3.5 (4.1)	4.3 (4.7)	16.7	3.5 (4.1)	3.8 (3.5)	6.4
Days of neurological support, mean (SD)	3.4 (7.0)	2.4 (5.3)	15.3	3.4 (7.0)	3.0 (6.0)	5.4
Days of renal support, mean (SD)	1.4 (3.9)	2.3 (6.6)	16.2	1.4 (3.9)	1.0 (3.3)	10.2
Receiving sedatives on final day, n (%)	2 (2.7)	92 (9.2)	27.7	2 (2.7)	3 (4.1)	7.4
Advanced respiratory support on final day, n (%)	25 (33.8)	322 (32.2)	3.3	25 (33.8)	16 (21.6)	27.2
Basic respiratory support on final day, n (%)	43 (58.1)	612 (61.3)	6.4	43 (58.1)	50 (67.6)	19.5
Circulatory support on final day, n (%)	0 (0)	32 (3.2)	25.7	0 (0)	0 (0)	N/A
Neurological support on final day, n (%)	0 (0)	22 (2.2)	21.2	0 (0)	0 (0)	N/A

Renal support on final day, n (%)	0 (0)	37 (3.7)	27.7	0 (0)	0 (0)	N/A

[a]999 days of observation from 294 patients; [b]52 unique patients after matching with replacement

APACHE, Acute Physiology And Chronic Health Evaluation; APS, Acute Physiology Score; SD, standard deviation
