# Supplementary material for: Does delaying discharge from intensive care until after tracheostomy removal affect 30-day mortality? Propensity score matched cohort study
Source: BMJ Open. 2020 Jun 7;10(6):e037762. doi: 10.1136/bmjopen-2020-037762 (PMC7282394; doi:10.1136/bmjopen-2020-037762)
Supplement: Author's manuscript [file bmjopen-2020-037762.draft_revisions.pdf]

BMJ Open is committed to open peer review. As part of this commitment we make the peer review history of every article we publish publicly available.

When an article is published we post the peer reviewers' comments and the authors' responses online. We also post the versions of the paper that were used during peer review. These are the versions that the peer review comments apply to.

The versions of the paper that follow are the versions that were submitted during the peer review process. They are not the versions of record or the final published versions. They should not be cited or distributed as the published version of this manuscript.

BMJ Open is an open access journal and the full, final, typeset and author-corrected version of record of the manuscript is available on our site with no access controls, subscription charges or pay-per-view fees (http://bmjopen.bmj.com).

If you have any questions on BMJ Open's open peer review process please email info.bmjopen@bmj.com

BMJ Open

Does delaying discharge from intensive care until after tracheostomy removal affect 30-day mortality? A propensity score matched cohort study.

Journal:	*BMJ Open*
Manuscript ID	bmjopen-2020-037762
Article Type:	Original research
Date Submitted by the Author:	14-Feb-2020
Complete List of Authors:	Vollam, Sarah; University of Oxford, Nuffield Department of Neurosciences<br>Harrison, David; ICNARC,<br>Young, Duncan; Oxford University, Nuffield Department of Clinical Neurosciences<br>Watkinson, Peter; Oxford University Hospitals NHS Trust, Kadoorie Centre for Critical Care research and Education
Keywords:	Adult intensive & critical care < ANAESTHETICS, RESPIRATORY MEDICINE (see Thoracic Medicine), Organisation of health services < HEALTH SERVICES ADMINISTRATION & MANAGEMENT

SCHOLARONE™
Manuscripts

I, the Submitting Author has the right to grant and does grant on behalf of all authors of the Work (as defined in the below author licence), an exclusive licence and/or a non-exclusive licence for contributions from authors who are: i) UK Crown employees; ii) where BMJ has agreed a CC-BY licence shall apply, and/or iii) in accordance with the terms applicable for US Federal Government officers or employees acting as part of their official duties; on a worldwide, perpetual, irrevocable, royalty-free basis to BMJ Publishing Group Ltd ("BMJ") its licensees and where the relevant Journal is co-owned by BMJ to the co-owners of the Journal, to publish the Work in this journal and any other BMJ products and to exploit all rights, as set out in our licence.

The Submitting Author accepts and understands that any supply made under these terms is made by BMJ to the Submitting Author unless you are acting as an employee on behalf of your employer or a postgraduate student of an affiliated institution which is paying any applicable article publishing charge ("APC") for Open Access articles. Where the Submitting Author wishes to make the Work available on an Open Access basis (and intends to pay the relevant APC), the terms of reuse of such Open Access shall be governed by a Creative Commons licence – details of these licences and which Creative Commons licence will apply to this Work are set out in our licence referred to above.

Other than as permitted in any relevant BMJ Author's Self Archiving Policies, I confirm this Work has not been accepted for publication elsewhere, is not being considered for publication elsewhere and does not duplicate material already published. I confirm all authors consent to publication of this Work and authorise the granting of this licence.

For peer review only

1

Does delaying discharge from intensive care until after tracheostomy removal affect 30-day mortality? A propensity score matched cohort study.

Mrs Sarah A Vollam (MSc) (Corresponding author)
Nurse Researcher

Nuffield Department of Clinical Neurosciences

University of Oxford

Kadoorie Centre for Critical Care Reseach and Education

Level 3, John Radcliffe Hospital

Oxford

OX3 9DU

sarah.vollam@ndcn.ox.ac.uk

Prof David A Harrison (PhD)
Senior Statistician

Intensive Care National Audit and Research Centre (ICNARC)

London, UK

Prof J Duncan Young (MD)
Professor of Intensive Care Medicine

Nuffield Department of Clinical Neurosciences, University of Oxford

Oxford, UK

Assoc Prof Peter J Watkinson (MD)
Consultant and Associate Professor in Intensive Care Medicine

Nuffield Department of Clinical Neurosciences, University of Oxford

Oxford, UK

WORD COUNT: 2,706

Keywords:

Tracheostomy Intensive Care Mortality

Decannulation Propensity Score

For peer review only - http://bmjopen.bmj.com/site/about/guidelines.xhtml

ABSTRACT

Objectives

To investigate the short-term mortality effect of discharge from an Intensive Care Unit (ICU) with a tracheostomy in place in comparison to delaying discharge until after tracheostomy removal.

Design

A propensity score matched cohort study using data from seventy-two United Kingdom ICUs taking part in the TracMan study. We matched patients discharged with a tracheostomy in place 1:1 with patients who remained in an ICU until either their tracheostomy was removed or they died with the tracheostomy in place. Propensity models were developed according to discharge destination, accounting for likely confounding factors. The primary outcome was 30-day mortality from matching day.

Results

622 patients received a tracheostomy whilst in the TracMan study. 144 patients left ICU with a tracheostomy. 999 days of observation from 294 patients were included in the control pool. 22 [15%] patients who left ICU with a tracheostomy died within 30 days compared to 26 [18%] who remained in ICU (RR 0.98, 95% CI 0.43, 2.23).

Conclusion

Keeping patients on an ICU to provide tracheostomy care was not found to affect mortality. Tracheostomy presence may indicate a higher risk of mortality due to underlying diseases and conditions rather than posing a risk in itself.

Strengths and Limitations

- The data used come from a clinical trial, using strategies to ensure a robust data set for analysis.

For peer review only - http://bmjopen.bmj.com/site/about/guidelines.xhtml

- The Trachman study was closed in 2008, limiting generalisability with current practice.
- Even with a database of 909 patients from a 72-centre study of tracheostomy timing we were only able to generate 144 cases, limiting the power of the study to detect an effect of remaining on an ICU for tracheostomy care.
- 622 patients were included in the TracMan trial, allowing a large pool of participants from multiple sites and a close match for almost all patients.
- Despite limitations, the TracMan database is a rich source from which to further our knowledge about patients who receive a tracheostomy whilst on an ICU and we are not aware of a similar prospective database.

For peer review only

4

INTRODUCTION

Patients who need prolonged artificial ventilation on an ICU commonly have a tracheostomy placed.[1-4] Perceived benefits include decreased sedation use, with increased patient comfort and mobility.[5] In some hospitals patients who no longer require a ventilator but still need a tracheostomy stay on an ICU until the tracheostomy is removed. In other hospitals patients with a tracheostomy leave ICU and receive care on general wards.

Patients leaving an ICU with a tracheostomy have higher in-hospital mortality than those discharged without a tracheostomy.[1, 6, 7] A tracheostomy may simply be a marker of a group of patients at greater risk of death from other causes (including the illness or condition that necessitates the tracheostomy). Alternatively, ward tracheostomy care may increase mortality (because for example there is insufficient skilled care to avoid direct tracheostomy-related deaths). Discovering which of these explains the high in-hospital mortality rates would change practice. Only discharging patients to the ward after decannulation would reduce mortality if ward tracheostomy care is insufficient. This would need a substantial change in current practice. If the high mortality is caused by other factors associated with the continued tracheostomy requirement, interventions should target these factors. A randomised, controlled study where half the patients are kept on an ICU until decannulation would resolve this question. However the costs of such a study would be very high as ICU bed capacity would have to increase to accommodate the intervention. A study using propensity score matching to investigate this approach is therefore a compelling first step.

Seventy-two ICUs in the UK took part in the TracMan randomised controlled trial.[8] The trial compared early tracheostomy (within 4 days) with deferred tracheostomy (after 10 days if still indicated). The study generated a tightly-defined, detailed database. This allowed us to undertake a propensity score matched cohort study. We investigated the effect of delaying discharge from an ICU until after tracheostomy removal (or death) in comparison to discharge from ICU with a tracheostomy in place.

For peer review only - http://bmjopen.bmj.com/site/about/guidelines.xhtml

5

MATERIALS AND METHODS

Data

We extracted information from the TracMan trial database. Nine hundred and nine adult patients from 72 ICUs in the United Kingdom participated. Written, informed consent or signed consultee agreement from the patients' legal representative/welfare guardian was obtained. The study was approved by Scotland A REC, reference 04/MRE00/43. All participants had received artificial ventilation for less than 4 days at study entry. The lead clinician predicted they would need at least 7 more days of artificial ventilation. Detailed inclusion criteria and other study details are published.[8] There were no missing data. The TracMan trial was registered on the ISRCTN database (ISRCTN28588190). Patient and Public Involvement was not sought for this study using a clinical trial database.

Definitions

We included patients who received a tracheostomy in either arm of the TracMan study. We excluded patients randomised in error or withdrawn from the trial before 30 days. We also excluded patients transferred to another ICU or discharged to another hospital or home. We defined two groups. The "discharged with a tracheostomy" group had left an ICU with a tracheostomy in place. They went to a ward or a high dependency unit (HDU) in the same hospital. The "remained in ICU" group had either been decannulated before they left the ICU or died in the ICU with the tracheostomy in place.

Development of propensity models

We chose propensity matching to produce a "remained in ICU" population as similar as possible to the population "discharged with a tracheostomy". This prioritised matching the populations above the individual matching of patients (as would occur with individual patient matching). We developed

For peer review only - http://bmjopen.bmj.com/site/about/guidelines.xhtml

two propensity models using logistic regression. One model was for discharge to a general ward with a tracheostomy in place. The other model was for discharge to an HDU with a tracheostomy in place. We chose potential confounders from those available within the TracMan dataset. The original TracMan dataset was chosen by an expert trial team and underwent stringent external review. We undertook a literature review to inform the choices made. The following variables were included in the propensity model (as potential confounders of the relationship between leaving an ICU with a tracheostomy in place and short term mortality):

- Age on admission to the ICU
- APACHE II Acute Physiology Score on ICU admission
- Surgical status (admission from an operating theatre following elective/scheduled surgery; admission from theatre following emergency/urgent surgery; non-surgical)
- Underlying reason for mechanical ventilation (neurological/non-neurological)
- Number of days since tracheostomy (up to and including the day of discharge for cases, or matched day for controls)
- Number of days receiving an intravenous bolus dose or an infusion of drugs primarily for sedation (up to and including the day of discharge for cases, or matched day for matched controls)
- Receiving an intravenous bolus dose or an infusion of drugs primarily for sedation on the day of discharge for cases, or matched day for matched controls
- Number of days of support (up to and including the day of discharge for cases, or matched day for matched controls) of the following organ systems:
    - Advanced respiratory support (invasive artificial ventilation; extracorporeal gas exchange)
    - Basic respiratory support (mask continuous positive airway pressure [CPAP]; non-invasive ventilation; >50% inspired oxygen; recent extubation following prolonged

For peer review only - http://bmjopen.bmj.com/site/about/guidelines.xhtml

mechanical ventilation; intubation to protect the airway; physiotherapy to clear secretions at least two hourly)

    - Circulatory support (vasoactive drug infusion; circulatory instability due to hypovolaemia; patients resuscitated following cardiac arrest; intra-aortic balloon pump in place)
    - Neurological support (central nervous system depression sufficient to prejudice the airway; invasive neurological monitoring)
    - Renal support (acute renal replacement therapy).
- Organ support (as above) received on the day of discharge for cases, or matched day for controls.

Matching

We matched patients discharged with a tracheostomy (cases) 1:1 with the patient (and day) from the “remained in ICU” group (controls). We matched to the patient with the closest propensity score (predicted log odds from the propensity model). We matched patients up to a difference of 1 standard deviation of the propensity score. We used the ward or HDU propensity model according to the patient’s discharge destination.

To ensure comparability between the two groups, the “remained in ICU” group were required to: have had the tracheostomy for at least two days by the matched day; have not received advanced respiratory support on the day following the matched day; and (for controls matched to patients discharged to a ward with the tracheostomy) have not received neurological support or renal support on the day following the matched day. We give our definitions of organ support below.

Outcome

The primary outcome was 30-day mortality from the matching day. For the “discharged with a tracheostomy” group this was death within 30 days after the discharge day. For the “remained in ICU” group, this was death within 30 days after the matched day.

For peer review only - http://bmjopen.bmj.com/site/about/guidelines.xhtml

Assessment of balance

We compared cases and controls before and after matching, for their age, sex, APACHE II Acute Physiology Score, overall APACHE II Score, surgical status, underlying reason for mechanical ventilation, number of days since tracheostomy, number of days receiving sedatives, number of days of organ support, and organ support received on matched day. We compared continuous variables with mean and standard deviation. We compared categorical variables with number and percent. We compared balance with the standardised difference. We defined the standardised difference as the absolute value of the difference in means divided by the pooled standard deviation.[9] In addition, we used quantile-quantile (QQ) plots to assess the balance across the full distribution for age, APACHE II score and propensity score. We assessed balance for the full cohort and split by the case's destination following discharge (ward or HDU).

Statistical Analysis

We estimated the relative risk of 30-day mortality for cases compared with matched controls by Poisson regression, conditional on the matched data.[10] We estimated standard errors with the non-parametric bootstrap.[11] We calculated relative risks both unadjusted and adjusted for age, APACHE II Acute Physiology Score and respiratory support on the matched day, to control for any remaining imbalance in these risk factors. We evaluated any difference in effect between patients discharged to a ward and those discharged to an HDU by introducing an interaction term in the Poisson regression model. We performed all analyses using Stata/SE Version 13.0 (StataCorp LP, College Station, Texas, USA).

RESULTS

909 patients were enrolled in the TracMan trial between November 2004 and November 2008. We excluded 10 patients randomised in error or withdrawn from the trial before 30 days. Of the remaining 899 patients, 622 (69.2%) received a tracheostomy. 174 deaths in ICU occurred in this

For peer review only - http://bmjopen.bmj.com/site/about/guidelines.xhtml

group. 144 patients had a tracheostomy in place when they left an ICU. 70 (11.3%) were discharged to a ward in the same hospital. 74 (11.9%) were discharged to an HDU in the same hospital. We included 999 days of observation from 294 patients in the control pool. For the ward control pool we included 960 days of observation from 292 patients (as we did not include days where patients received neurological or renal support). Figure 1 shows the selection process for cases and controls.

Table 1 shows balance statistics for the full cohort, before and after matching. Balance statistics for those discharged to a ward and those discharged to an HDU are included in the supplementary material – Tables S1 and S2. There was imbalance in many covariates before matching (as indicated by standardised difference values above 10). Balance improved after matching. Figure 2 shows QQ plots for age, APACHE II score and propensity score. The QQ plot of propensity score balance shows almost all patients were closely matched.

Remaining in an ICU for tracheostomy care did not statistically affect mortality in comparison to care outside an ICU (Table 2).

For peer review only

Table 1 Balance in potential confounders before and after matching – full cohort

	Before matching			After matching		
	Discharged with tracheostomy (N=144)	Remained in ICU (N=999[a])	Standardised difference	Discharged with tracheostomy (N=144)	Remained in ICU (N=144[b])	Standardised difference
Age (years), mean (SD)	64.5 (12.5)	62.7 (13.1)	14.1	64.5 (12.5)	65.4 (11.2)	7.2
Male, n (%)	82 (56.9)	644 (64.5)	15.4	82 (56.9)	104 (72.2)	32.2
APACHE II APS, mean (SD)	14.4 (5.8)	13.4 (6.5)	16.2	14.4 (5.8)	13.7 (6.7)	11.4
APACHE II Score, mean (SD)	19.2 (6.8)	17.9 (7.0)	19.3	19.2 (6.8)	18.8 (7.3)	5.6
Surgical status – elective/scheduled, n (%)	8 (5.6)	51 (5.1)	2.0	8 (5.6)	10 (6.9)	5.7
Surgical status – emergency/urgent, n (%)	24 (16.7)	140 (14.0)	7.4	24 (16.7)	25 (17.4)	1.8
Neurological reason for ventilation, n (%)	18 (12.5)	100 (10.0)	7.9	18 (12.5)	19 (13.2)	2.1
Days since tracheostomy, mean (SD)	14.6 (10.4)	17.2 (13.6)	21.4	14.6 (10.4)	14.1 (10.9)	4.6
Days receiving sedatives, mean (SD)	8.1 (6.9)	9.1 (6.9)	14.6	8.1 (6.9)	7.8 (5.7)	4.6
Days of advanced respiratory support, mean (SD)	15.3 (10.9)	19.0 (14.1)	29.2	15.3 (10.9)	15.1 (11.9)	1.8
Days of basic respiratory support, mean (SD)	5.9 (9.5)	5.5 (9.5)	4.5	5.9 (9.5)	6.8 (11.9)	8.3
Days of circulatory support, mean (SD)	3.4 (3.7)	4.3 (4.7)	21.2	3.4 (3.7)	3.2 (3.2)	5.0
Days of neurological support, mean (SD)	3.5 (6.6)	2.4 (5.3)	18.9	3.5 (6.6)	3.0 (5.2)	10.0
Days of renal support, mean (SD)	1.2 (4.2)	2.3 (6.6)	19.9	1.2 (4.2)	0.9 (3.0)	8.8
Receiving sedatives on final day, n (%)	8 (5.6)	92 (9.2)	14.0	8 (5.6)	7 (4.9)	3.1
Advanced respiratory support on final day, n (%)	29 (20.1)	322 (32.2)	27.7	29 (20.1)	21 (14.6)	14.7
Basic respiratory support on final day, n (%)	89 (61.8)	612 (61.3)	1.1	89 (61.8)	90 (62.5)	1.4
Circulatory support on final day, n (%)	0 (0)	32 (3.2)	25.7	0 (0)	0 (0)	N/A
Neurological support on final day, n (%)	6 (4.2)	22 (2.2)	11.2	6 (4.2)	2 (1.4)	16.9
Renal support on final day, n (%)	0 (0)	37 (3.7)	27.7	0 (0)	0 (0)	N/A

[a]999 days of observation from 294 patients; [b]84 unique patients after matching with replacement

APACHE, Acute Physiology And Chronic Health Evaluation; APS, Acute Physiology Score; SD, standard deviation

For peer review only - http://bmjopen.bmj.com/site/about/guidelines.xhtml

Table 2 Outcome of 30-day mortality following discharge with a tracheostomy in place compared with those who remained in an ICU

	30-day mortality, deaths (%) [95% CI]		Unadjusted RR [95% CI]	P value	Adjusted RR[a] [95% CI]	P value
	Discharged with tracheostomy	Remained in ICU				
Overall (N=144)	22 (15.3) [10.3, 22.0]	26 (18.1) [12.6, 25.1]	0.85 [0.49, 1.47]	0.55	0.98 [0.43, 2.23]	0.96
Discharged to a ward (N=70)	13 (18.6) [11.2, 29.2]	11 (15.7) [9.0, 26.0]	1.18 [0.48, 2.89]	0.48[b]	1.46 [0.45, 4.78]	0.37[b]
Discharged to an HDU (N=74)	9 (12.2) [6.5, 21.5]	15 (20.3) [12.7, 30.8]	0.60 [0.11, 3.30]		0.61 [0.15, 2.57]	

[a]Adjusted for age and APACHE II Acute Physiology Score and respiratory support on final day; [b]P value for test of interaction with destination following discharge

CI, confidence interval; RR, relative risk

For peer review only

DISCUSSION

There is little doubt that hospital mortality after leaving an ICU is high in patients with a new tracheostomy.[1, 2, 7] Our study supports these previous findings. Patients leaving ICU with a tracheostomy had a 30 day mortality nearly twice the 9.5% in-hospital mortality for all patients discharged from UK ICUs at the time.[12] There are two possible explanations for this high mortality. There may be deficiencies in tracheostomy care outside ICU, which staying in an ICU would avoid. Alternatively, having a tracheostomy when leaving an ICU may simply mark a higher risk group. To separate these two possibilities, we propensity score matched patients who left an ICU with a tracheostomy with patients who remained in an ICU with a tracheostomy. The TracMan database contained high quality daily patient information. These data allowed us to match on time and treatment periods as well as static variables. Patients required similar levels of support when matched. We found no significant difference in post-ICU 30-day mortality. This remained true when we separated patients discharged to a general ward and those discharged to an HDU.

Our propensity score matched cohort design limits the study findings. Although balance was improved between groups after matching, imbalances remained. This was always likely given the number of factors matched. Our results may be confounded by an unknown and unrecorded variable that alters both the likelihood of leaving an ICU with a tracheostomy and subsequent death. This is a risk in all similarly designed studies. Our detailed database, allowing us to match on multiple variables, should have minimised this risk.

The data used come from a clinical trial. Variables were collected using a comprehensive set of definitions to ensure consistency. Out-of-range or missing data were checked during the trial with source documents. These strategies provided a robust data set for analysis. However, the database does not include all patients who had a tracheostomy at the centres during the study period. Some patients will have received a tracheostomy outside the study. Patients in a clinical trial may experience closer monitoring, and therefore different care, than in usual practice.[13] However, the

For peer review only - http://bmjopen.bmj.com/site/about/guidelines.xhtml

TracMan study did not require ward or High Dependency Unit follow-up by research staff. The risk that researcher staff directly affected ward care appears small.

Even with a 909-patient database available from a 72-centre study of tracheostomy timing we were only able to generate 144 cases, limiting the power of the study to detect an effect of remaining on an ICU for tracheostomy care. The wide confidence intervals around our estimates mean we cannot exclude a clinically relevant effect. Cases were matched 1:1 with controls. Matching at one case to two controls may have increased power slightly. Conversely, as we used replacement this would also have increased the duplication of patients in the control group.

Although these concerns place some limitations on the generalisability of our study, the results remain important. Despite being over 10 years old, the TracMan database is a rich source from which to further our knowledge about patients who receive a tracheostomy whilst on an ICU. We are not aware of a similar prospective database. Our study benefits from the large number (622) of patients who underwent a tracheostomy within the TracMan trial, and the prospective daily information gathered. These factors allowed a large pool of patients from which to match. As a result, almost all patients were closely matched. The scale of the database allowed us to include substantially more patients from many more centres than has previously been possible.

A previous multi-centre study took a different propensity-based approach. They compared 141 patients leaving an ICU with a tracheostomy in place with 60 leaving after tracheostomy removal.[2] No difference in hospital mortality *after leaving* an ICU was found. Our study assesses the effect of tracheostomy care *in an ICU* in comparison to care outside an ICU at an equivalent point in patients' recovery. Our approach has the advantage that mortality whilst on ICU receiving tracheostomy care is taken into account. Neither approach suggests that mortality is increased by tracheostomy care outside an ICU. The two propensity-based studies have findings that contrast with previous research.[6, 7] The findings demonstrate the importance of taking imbalances in other risk factors into account.

For peer review only - http://bmjopen.bmj.com/site/about/guidelines.xhtml

Two other information sources support the view that the mortality attributable to the tracheostomy and its care after leaving an ICU may be low. A study reviewing 453 incidents related to tracheostomies in patients treated on hospital wards reported to the UK National Patient Safety Agency over two years[14] found only 15 (3%) where the incident was associated with the death of a patient. In a national audit only 5 of 518 (1%) ward patients with a tracheostomy were classified as having suffered harm from tracheostomy-related problems. Only one of these suffered a cardiac arrest.[15]

The question of where to provide care for patients who have had a tracheostomy placed on an ICU might best be answered by a randomised controlled trial. However, the incidence of events in our study and those of others suggest a very large (and so expensive) trial would be required.[2] At present, a propensity-matched approach may provide the highest level of evidence practically available.

Recommendations for improving ward outcomes for patients leaving an ICU with a tracheostomy have focused on tracheostomy care.[15-17] Ensuring appropriate tracheostomy care and training for tracheostomy-related emergencies is clearly important. Other problems underlie the continued need for a tracheostomy on the ward. Our findings suggest that mortality may be more related to the underlying diseases and conditions than the tracheostomy itself. We need better understanding of these issues to improve outcomes for these vulnerable patients.

For peer review only

CONCLUSION

In a propensity-based analysis of a large prospective dataset, 30-day mortality was high in patients who could be discharged from an ICU with a tracheostomy in place. However, keeping patients on an ICU to provide tracheostomy care did not affect mortality in comparison to ward-based care. Tracheostomy presence may indicate a higher risk of mortality due to underlying diseases and

For peer review only - http://bmjopen.bmj.com/site/about/guidelines.xhtml

conditions rather than posing a risk in itself. This should be considered when planning care for patients with a tracheostomy.

ACKNOWLEDGEMENTS

The full list of TracMan trial collaborators has been published and their major contribution to this study is acknowledged. The TracMan trial was registered on the ISRCTN database (ISRCTN28588190).

FUNDING

The work described in this paper was funded by the National Institute for Health Research (NIHR) Oxford Biomedical Research Centre (BRC) and by the Intensive Care National Audit and Research Centre. The views expressed are those of the author(s) and not necessarily those of the NHS, the NIHR or the Department of Health. The original TracMan study was funded by the UK Intensive Care Society and the Medical Research Council.

DECLARATION OF COMPETING INTERESTS

Peter Watkinson co-developed the System for Electronic Notification and Documentation (SEND), for which Drayson Health has purchased a sole licence. The company has a research agreement with the University of Oxford and royalty agreements with Oxford University Hospitals NHS Trust and the University of Oxford. Drayson Health may in the future pay Peter Watkinson personal fees.

CONTRIBUTION STATEMENT

DY, PW and SV conceived the original idea. SV and DY prepared the manuscript with revisions from PW and DH. All authors read and approved the final manuscript. All authors had full access to all of the data (including statistical reports and tables) in the study and take responsibility for the integrity of the data and the accuracy of the data analysis.

DATA SHARING STATEMENT

Deidentified data may be made available on reasonable request to the corresponding author.

For peer review only - http://bmjopen.bmj.com/site/about/guidelines.xhtml

For peer review only

REFERENCES

[1] Clec'h C, Alberti C, Vincent F, et al. Tracheostomy does not improve the outcome of patients requiring prolonged mechanical ventilation: a propensity analysis. *Crit Care Med* 2007;35:132-8.

[2] Fernandez R, Tizon AI, Gonzalez J, et al. Intensive care unit discharge to the ward with a tracheostomy cannula as a risk factor for mortality: a prospective, multicenter propensity analysis. *Crit Care Med* 2011;39:2240-5.

[3] Nathens AB, Rivara FP, Mack CD, et al. Variations in rates of tracheostomy in the critically ill trauma patient. *Crit Care Med* 2006;34:2919-24.

[4] Mehta AB, Syeda SN, Bajpayee L, et al. Trends in Tracheostomy for Mechanically Ventilated Patients in the United States, 1993-2012. *AJRCCM* 2015;192:446-54.

[5] Freeman BD, Morris PE. Tracheostomy practice in adults with acute respiratory failure. *Crit Care Med* 2012;40:2890-6.

[6] Araujo I, Goncalves-Pereira J, Teixeira S, et al. Assessment of risk factors for in-hospital mortality after intensive care unit discharge. *Biomarkers* 2012;17:180-5.

[7] Fernandez R, Bacelar N, Hernandez G, et al. Ward mortality in patients discharged from the ICU with tracheostomy may depend on patient's vulnerability. *Intensive Care Med* 2008;34:1878-82.

[8] Young D, Harrison DA, Cuthbertson BH, et al. Effect of early vs late tracheostomy placement on survival in patients receiving mechanical ventilation: the TracMan randomized trial. *JAMA* 2013;309(20):2121-9.

[9] Austin PC. Balance diagnostics for comparing the distribution of baseline covariates between treatment groups in propensity-score matched samples. *Stat Med* 2009;28(25):3083-107.

[10] Cummings PBM. Analysis of matched cohort data. *Stata J* 2004;4:274-81.

[11] Cummings P, McKnight B, Greenland S. Matched cohort methods for injury research. *Epidemiol Rev* 2003;25:43-50.

For peer review only - http://bmjopen.bmj.com/site/about/guidelines.xhtml

17

[12] ICNARC: Intensive Care National Audit & Rresearch Centre (ICNARC). Case Mix Programme Summary Statistics 2008-09. www.icnarc.org/Our-Audit/Audits/Cmp/Reports/Summary-Statistics. (Acessed 14/02/2020).

[13] McCarney R, Warner J, Iliffe S, et al. The Hawthorne Effect: a randomised, controlled trial. *BMC Med Res Methodol* 2007;7:30.

[14] McGrath BA, Thomas AN. Patient safety incidents associated with tracheostomies occurring in hospital wards: a review of reports to the UK National Patient Safety Agency. *Postgrad Med J* 2010; 86:522-5.

[15] Wilkinson KA, Martin IC, Freeth H, et al. On the Right Trach? A review of the care received by patients who underwent a tracheostomy. A report by the National Confidential Enquiry into Patient Outcome and Death. 2014.

[16] The global tracheostomy collaborative. http://globaltrach.org/. (Accessed 14/02/2020).

[17] National Tracheostomy Safety Project. www.tracheostomy.org.uk. (Accessed 14/02/2020).

Figure legends:

Figure 1: Case and control selection flow diagram

Figure 2: Quantile-quantile (QQ) plots of balance in (A) age, (B) APACHE II Acute Physiology Score and (C) propensity score

For peer review only

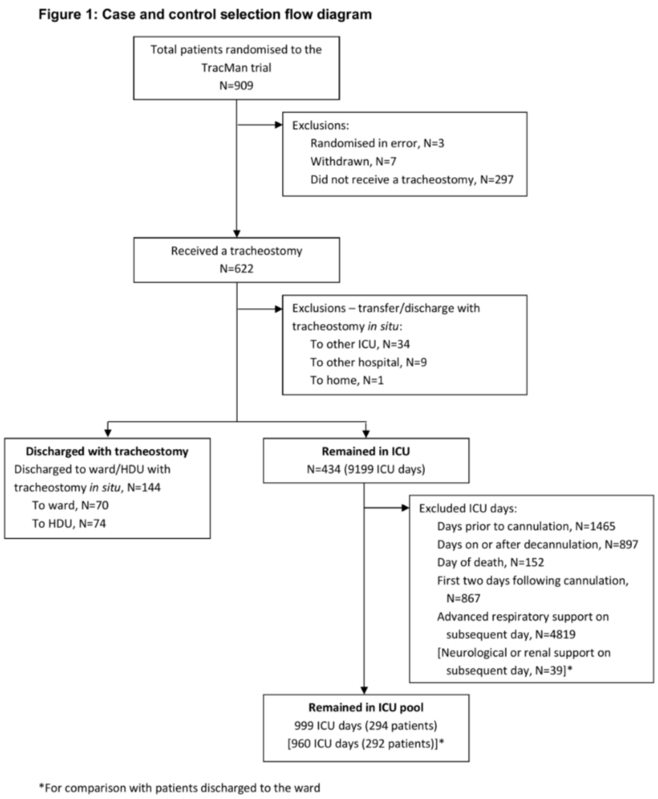

Figure 1: Case and control selection flow diagram

Figure 2 Quantile-quantile (QQ) plots of balance in (A) age, (B) APACHE II Acute Physiology Score and (C) propensity score

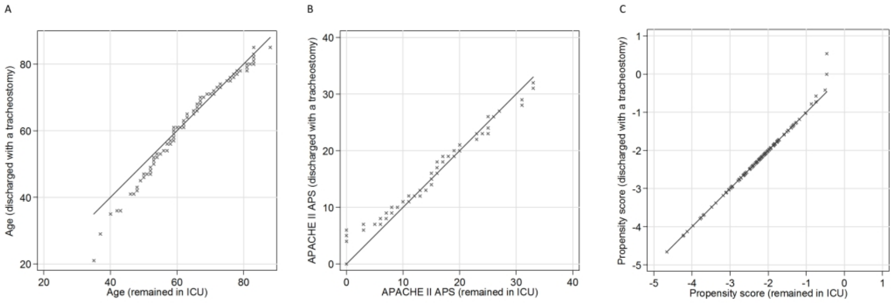

Figure 2: Quantile-quantile (QQ) plots of balance in (A) age, (B) APACHE II Acute Physiology Score and (C) propensity score

SUPPLEMENTARY MATERIAL: Balance in potential confounders

Table S1 Balance in potential confounders before and after matching – cases discharged to a ward

Potential confounder	Before matching			After matching		
	Discharged with tracheostomy (N=70)	Remained in ICU (N=960[a])	Standardised difference	Discharged with tracheostomy (N=70)	Remained in ICU (N=70[b])	Standardised difference
Age (years), mean (SD)	65.0 (13.3)	63.3 (12.9)	13.1	65.0 (13.3)	66.3 (10.3)	10.9
Male, n (%)	50 (71.4)	607 (63.2)	17.5	50 (71.4)	48 (68.6)	6.2
APACHE II APS, mean (SD)	14.7 (6.2)	13.2 (6.5)	25.0	14.7 (6.2)	14.9 (6.7)	3.3
APACHE II Score, mean (SD)	19.5 (7.1)	17.7 (7.0)	25.8	19.5 (7.1)	20.0 (7.4)	6.7
Surgical status – elective/scheduled, n (%)	2 (2.9)	51 (5.3)	12.4	2 (2.9)	2 (2.9)	0
Surgical status – emergency/urgent, n (%)	10 (14.3)	139 (14.5)	0.5	10 (14.3)	10 (14.3)	0
Neurological reason for ventilation, n (%)	11 (15.7)	94 (9.8)	17.7	11 (15.7)	10 (14.3)	4.0
Days since tracheostomy, mean (SD)	15.7 (11.0)	17.2 (13.7)	11.8	15.7 (11.0)	15.5 (11.8)	1.5
Days receiving sedatives, mean (SD)	7.6 (5.1)	8.9 (6.7)	23.1	7.6 (5.1)	7.5 (5.3)	1.6
Days of advanced respiratory support, mean (SD)	14.7 (9.4)	19.1 (14.3)	36.0	14.7 (9.4)	14.6 (12.3)	0.9
Days of basic respiratory support, mean (SD)	6.1 (7.7)	5.6 (9.7)	6.0	6.1 (7.7)	6.3 (11.0)	1.8
Days of circulatory support, mean (SD)	3.2 (3.4)	4.1 (4.6)	23.5	3.2 (3.4)	2.6 (2.7)	20.0
Days of neurological support, mean (SD)	3.7 (6.2)	2.3 (5.1)	26.1	3.7 (6.2)	2.9 (4.3)	16.0
Days of renal support, mean (SD)	0.9 (4.6)	1.6 (5.4)	14.1	0.9 (4.6)	0.7 (2.6)	7.3
Receiving sedatives on final day, n (%)	6 (8.6)	83 (8.6)	0.3	6 (8.6)	4 (5.7)	11.0
Advanced respiratory support on final day, n (%)	4 (5.7)	310 (32.3)	71.9	4 (5.7)	5 (7.1)	5.8
Basic respiratory support on final day, n (%)	46 (65.7)	593 (61.8)	8.2	46 (65.7)	40 (57.1)	17.6
Circulatory support on final day, n (%)	0 (0)	32 (3.3)	26.2	0 (0)	0 (0)	N/A
Neurological support on final day, n (%)	6 (8.6)	11 (1.1)	34.8	6 (8.6)	2 (2.9)	24.6
Renal support on final day, n (%)	0 (0)	8 (0.8)	13.0	0 (0)	0 (0)	N/A

For peer review only

[a]960 days of observation from 292 patients; [b]49 unique patients after matching with replacement

APACHE, Acute Physiology And Chronic Health Evaluation; APS, Acute Physiology Score; SD, standard deviation

Table S2 Balance in potential confounders before and after matching – cases discharged to an HDU

Potential confounder	Before matching			After matching		
	Discharged with tracheostomy (N=74)	Remained in ICU (N=999[a])	Standardised difference	Discharged with tracheostomy (N=74)	Remained in ICU (N=74[b])	Standardised difference
Age (years), mean (SD)	64.1 (11.8)	62.7 (13.1)	11.0	64.1 (11.8)	64.5 (11.9)	3.5
Male, n (%)	32 (43.2)	644 (64.5)	43.4	32 (43.2)	56 (75.7)	69.5
APACHE II APS, mean (SD)	14.1 (5.5)	13.4 (6.5)	11.5	14.1 (5.5)	12.5 (6.5)	26.5
APACHE II Score, mean (SD)	18.9 (6.5)	17.9 (7.0)	15.5	18.9 (6.5)	17.7 (7.0)	18.3
Surgical status – elective/scheduled, n (%)	6 (8.1)	51 (5.1)	12.1	6 (8.1)	8 (10.8)	9.2
Surgical status – emergency/urgent, n (%)	14 (18.9)	140 (14.0)	13.2	14 (18.9)	15 (20.3)	3.4
Neurological reason for ventilation, n (%)	7 (9.5)	100 (10.0)	1.9	7 (9.5)	9 (12.2)	8.7
Days since tracheostomy, mean (SD)	13.6 (9.9)	17.2 (13.6)	30.5	13.6 (9.9)	12.8 (9.8)	7.9
Days receiving sedatives, mean (SD)	8.7 (8.2)	9.1 (6.9)	6.1	8.7 (8.2)	8.2 (6.0)	6.8
Days of advanced respiratory support, mean (SD)	15.9 (12.2)	19.0 (14.1)	23.5	15.9 (12.2)	15.6 (11.5)	2.5
Days of basic respiratory support, mean (SD)	5.8 (11.0)	5.5 (9.5)	2.7	5.8 (11.0)	7.4 (12..8)	13.2
Days of circulatory support, mean (SD)	3.5 (4.1)	4.3 (4.7)	16.7	3.5 (4.1)	3.8 (3.5)	6.4
Days of neurological support, mean (SD)	3.4 (7.0)	2.4 (5.3)	15.3	3.4 (7.0)	3.0 (6.0)	5.4
Days of renal support, mean (SD)	1.4 (3.9)	2.3 (6.6)	16.2	1.4 (3.9)	1.0 (3.3)	10.2
Receiving sedatives on final day, n (%)	2 (2.7)	92 (9.2)	27.7	2 (2.7)	3 (4.1)	7.4
Advanced respiratory support on final day, n (%)	25 (33.8)	322 (32.2)	3.3	25 (33.8)	16 (21.6)	27.2
Basic respiratory support on final day, n (%)	43 (58.1)	612 (61.3)	6.4	43 (58.1)	50 (67.6)	19.5
Circulatory support on final day, n (%)	0 (0)	32 (3.2)	25.7	0 (0)	0 (0)	N/A
Neurological support on final day, n (%)	0 (0)	22 (2.2)	21.2	0 (0)	0 (0)	N/A

For peer review only

Renal support on final day, n (%)	0 (0)	37 (3.7)	27.7	0 (0)	0 (0)	N/A

[a]999 days of observation from 294 patients; [b]52 unique patients after matching with replacement

APACHE, Acute Physiology And Chronic Health Evaluation; APS, Acute Physiology Score; SD, standard deviation

For peer review only

STROBE Statement—checklist of items that should be included in reports of observational studies

	Item No	Recommendation	Page
**Title and abstract**	1	(*a*) Indicate the study's design with a commonly used term in the title or the abstract	1
		(*b*) Provide in the abstract an informative and balanced summary of what was done and what was found	2
**Introduction**			
Background/rationale	2	Explain the scientific background and rationale for the investigation being reported	4
Objectives	3	State specific objectives, including any prespecified hypotheses	4
**Methods**			
Study design	4	Present key elements of study design early in the paper	5-6
Setting	5	Describe the setting, locations, and relevant dates, including periods of recruitment, exposure, follow-up, and data collection	5
Participants	6	(*a*) *Cohort study*—Give the eligibility criteria, and the sources and methods of selection of participants. Describe methods of follow-up<br>*Case-control study*—Give the eligibility criteria, and the sources and methods of case ascertainment and control selection. Give the rationale for the choice of cases and controls<br>*Cross-sectional study*—Give the eligibility criteria, and the sources and methods of selection of participants	5 – basic, detailed in other paper
		(*b*) *Cohort study*—For matched studies, give matching criteria and number of exposed and unexposed<br>*Case-control study*—For matched studies, give matching criteria and the number of controls per case	6-7
Variables	7	Clearly define all outcomes, exposures, predictors, potential confounders, and effect modifiers. Give diagnostic criteria, if applicable	4-7
Data sources/ measurement	8*	For each variable of interest, give sources of data and details of methods of assessment (measurement). Describe comparability of assessment methods if there is more than one group	5
Bias	9	Describe any efforts to address potential sources of bias	8
Study size	10	Explain how the study size was arrived at	n/a – whole cohort of previous study
Quantitative variables	11	Explain how quantitative variables were handled in the analyses. If applicable, describe which groupings were chosen and why	8
Statistical methods	12	(*a*) Describe all statistical methods, including those used to control for confounding	8
		(*b*) Describe any methods used to examine subgroups and interactions	8
		(*c*) Explain how missing data were addressed	4
		(*d*) *Cohort study*—If applicable, explain how loss to follow-up was addressed<br>*Case-control study*—If applicable, explain how matching of cases and controls was addressed<br>*Cross-sectional study*—If applicable, describe analytical methods taking account of sampling strategy	n/a
		(*e*) Describe any sensitivity analyses	8

Continued on next page

For peer review only - http://bmjopen.bmj.com/site/about/guidelines.xhtml

**Results**			
Participants	13*	(a) Report numbers of individuals at each stage of study—eg numbers potentially eligible, examined for eligibility, confirmed eligible, included in the study, completing follow-up, and analysed	8-9 and figure 1
		(b) Give reasons for non-participation at each stage	8-9 and figure 1
		(c) Consider use of a flow diagram	Figure 1
Descriptive data	14*	(a) Give characteristics of study participants (eg demographic, clinical, social) and information on exposures and potential confounders	Table 1
		(b) Indicate number of participants with missing data for each variable of interest	n/a
		(c) *Cohort study*—Summarise follow-up time (eg, average and total amount)	n/a
Outcome data	15*	*Cohort study*—Report numbers of outcome events or summary measures over time	
		Case-control study—Report numbers in each exposure category, or summary measures of exposure	Table 1
		Cross-sectional study—Report numbers of outcome events or summary measures	
Main results	16	(*a*) Give unadjusted estimates and, if applicable, confounder-adjusted estimates and their precision (eg, 95% confidence interval). Make clear which confounders were adjusted for and why they were included	Table 2
		(*b*) Report category boundaries when continuous variables were categorized	n/a
		(*c*) If relevant, consider translating estimates of relative risk into absolute risk for a meaningful time period	n/a
Other analyses	17	Report other analyses done—eg analyses of subgroups and interactions, and sensitivity analyses	Supp Tables 1 and 2 HDU/ward
**Discussion**			
Key results	18	Summarise key results with reference to study objectives	12
Limitations	19	Discuss limitations of the study, taking into account sources of potential bias or imprecision. Discuss both direction and magnitude of any potential bias	12-13
Interpretation	20	Give a cautious overall interpretation of results considering objectives, limitations, multiplicity of analyses, results from similar studies, and other relevant evidence	12
Generalisability	21	Discuss the generalisability (external validity) of the study results	13
**Other information**			
Funding	22	Give the source of funding and the role of the funders for the present study and, if applicable, for the original study on which the present article is based	15

For peer review only

BMJ Open

Does delaying discharge from intensive care until after tracheostomy removal affect 30-day mortality? A propensity score matched cohort study.

Journal:	*BMJ Open*
Manuscript ID	bmjopen-2020-037762.R1
Article Type:	Original research
Date Submitted by the Author:	14-Apr-2020

Complete List of Authors:	Vollam, Sarah; University of Oxford, Nuffield Department of Neurosciences<br>Harrison, David; ICNARC,<br>Young, Duncan; Oxford University, Nuffield Department of Clinical Neurosciences<br>Watkinson, Peter; Oxford University Hospitals NHS Trust, Kadoorie Centre for Critical Care research and Education

**Primary Subject Heading**:	Intensive care
Secondary Subject Heading:	Medical management, Evidence based practice, Health services research, Respiratory medicine
Keywords:	Adult intensive & critical care < ANAESTHETICS, RESPIRATORY MEDICINE (see Thoracic Medicine), Organisation of health services < HEALTH SERVICES ADMINISTRATION & MANAGEMENT

SCHOLARONE™
Manuscripts

I, the Submitting Author has the right to grant and does grant on behalf of all authors of the Work (as defined in the below author licence), an exclusive licence and/or a non-exclusive licence for contributions from authors who are: i) UK Crown employees; ii) where BMJ has agreed a CC-BY licence shall apply, and/or iii) in accordance with the terms applicable for US Federal Government officers or employees acting as part of their official duties; on a worldwide, perpetual, irrevocable, royalty-free basis to BMJ Publishing Group Ltd ("BMJ") its licensees and where the relevant Journal is co-owned by BMJ to the co-owners of the Journal, to publish the Work in this journal and any other BMJ products and to exploit all rights, as set out in our licence.

The Submitting Author accepts and understands that any supply made under these terms is made by BMJ to the Submitting Author unless you are acting as an employee on behalf of your employer or a postgraduate student of an affiliated institution which is paying any applicable article publishing charge ("APC") for Open Access articles. Where the Submitting Author wishes to make the Work available on an Open Access basis (and intends to pay the relevant APC), the terms of reuse of such Open Access shall be governed by a Creative Commons licence – details of these licences and which Creative Commons licence will apply to this Work are set out in our licence referred to above.

Other than as permitted in any relevant BMJ Author's Self Archiving Policies, I confirm this Work has not been accepted for publication elsewhere, is not being considered for publication elsewhere and does not duplicate material already published. I confirm all authors consent to publication of this Work and authorise the granting of this licence.

For peer review only

1

Does delaying discharge from intensive care until after tracheostomy removal affect 30-day mortality? A propensity score matched cohort study.

Mrs Sarah A Vollam (MSc) (Corresponding author)
Nurse Researcher

Nuffield Department of Clinical Neurosciences

University of Oxford

Kadoorie Centre for Critical Care Research and Education

Level 3, John Radcliffe Hospital

Oxford

OX3 9DU

sarah.vollam@ndcn.ox.ac.uk

Prof David A Harrison (PhD)
Senior Statistician

Intensive Care National Audit and Research Centre (ICNARC)

London, UK

Prof J Duncan Young (MD)
Professor of Intensive Care Medicine

Nuffield Department of Clinical Neurosciences, University of Oxford

Oxford, UK

Assoc Prof Peter J Watkinson (MD)
Consultant and Associate Professor in Intensive Care Medicine

Nuffield Department of Clinical Neurosciences, University of Oxford

Oxford, UK

WORD COUNT: 2,706

Keywords:

Tracheostomy Intensive Care Mortality

Decannulation Propensity Score

For peer review only

For peer review only - http://bmjopen.bmj.com/site/about/guidelines.xhtml

2

ABSTRACT

Objectives

To investigate the short-term mortality effect of discharge from an Intensive Care Unit (ICU) with a tracheostomy in place in comparison to delaying discharge until after tracheostomy removal.

Design

A propensity score matched cohort study using data from the TracMan study.

Setting

Seventy-two United Kingdom ICUs taking part in the TracMan study, a randomised controlled trial comparing early tracheostomy (within 4 days of critical care admission) with deferred tracheostomy (after 10 days if still indicated).

Participants

622 patients who received a tracheostomy whilst in the TracMan study between November 2004 and November 2008. 144 patients left ICU with a tracheostomy. 999 days of observation from 294 patients were included in the control pool.

Interventions

We matched patients discharged with a tracheostomy in place 1:1 with patients who remained in an ICU until either their tracheostomy was removed or they died with the tracheostomy in place. Propensity models were developed according to discharge destination, accounting for likely confounding factors.

Primary outcome measure

The primary outcome was 30-day mortality from the matching day. For the "discharged with a tracheostomy" group this was death within 30 days after the discharge day. For the "remained in ICU" group, this was death within 30 days after the matched day.

For peer review only - http://bmjopen.bmj.com/site/about/guidelines.xhtml

3

Results

22 [15%] patients who left ICU with a tracheostomy died within 30 days compared to 26 [18%] who remained in ICU (RR 0.98, 95% CI 0.43, 2.23).

Conclusion

Keeping patients on an ICU to provide tracheostomy care was not found to affect mortality. Tracheostomy presence may indicate a higher risk of mortality due to underlying diseases and conditions rather than posing a risk in itself.

Strengths and Limitations

- The data used come from a clinical trial, using strategies to ensure a robust data set for analysis.
- The Tracman study was closed in 2008, limiting generalisability with current practice.
- Even with a database of 909 patients from a 72-centre study of tracheostomy timing we were only able to generate 144 cases, limiting the power of the study to detect an effect of remaining on an ICU for tracheostomy care.
- 622 patients were included in the TracMan trial, allowing a large pool of participants from multiple sites and a close match for almost all patients.
- Despite limitations, the TracMan database is a rich source from which to further our knowledge about patients who receive a tracheostomy whilst on an ICU and we are not aware of a similar prospective database.

For peer review only - http://bmjopen.bmj.com/site/about/guidelines.xhtml

INTRODUCTION

Patients who need prolonged artificial ventilation on an ICU commonly have a tracheostomy placed.[1-4] Perceived benefits include decreased sedation use, with increased patient comfort and mobility.[5] In some hospitals patients who no longer require a ventilator but still need a tracheostomy stay on an ICU until the tracheostomy is removed. In other hospitals patients with a tracheostomy leave ICU and receive care on general wards.

Patients leaving an ICU with a tracheostomy have higher in-hospital mortality than those discharged without a tracheostomy.[1, 6, 7] A tracheostomy may simply be a marker of a group of patients at greater risk of death from other causes (including the illness or condition that necessitates the tracheostomy). Alternatively, ward tracheostomy care may increase mortality (because for example there is insufficient skilled care to avoid direct tracheostomy-related deaths). Discovering which of these explains the high in-hospital mortality rates would change practice. Only discharging patients to the ward after decannulation would reduce mortality if ward tracheostomy care is insufficient. This would need a substantial change in current practice. If the high mortality is caused by other factors associated with the continued tracheostomy requirement, interventions should target these factors. A randomised, controlled study where half the patients are kept on an ICU until decannulation would resolve this question. However the costs of such a study would be very high as ICU bed capacity would have to increase to accommodate the intervention. A study using propensity score matching to investigate this approach is therefore a compelling first step.

Seventy-two ICUs in the UK took part in the TracMan randomised controlled trial.[8] The trial compared early tracheostomy (within 4 days of critical care admission) with deferred tracheostomy (after 10 days if still indicated). The study generated a tightly-defined, detailed database. This allowed us to undertake a propensity score matched cohort study. We investigated the effect of delaying discharge from an ICU until after tracheostomy removal (or death) in comparison to discharge from ICU with a tracheostomy in place.

For peer review only - http://bmjopen.bmj.com/site/about/guidelines.xhtml

MATERIALS AND METHODS

Data

We extracted information from the TracMan trial database. Nine hundred and nine adult patients from 72 ICUs in the United Kingdom participated. Written, informed consent or signed consultee agreement from the patients' legal representative/welfare guardian was obtained. The study was approved by Scotland A REC, reference 04/MRE00/43. All participants had received artificial ventilation for less than 4 days at study entry. The lead clinician predicted they would need at least 7 more days of artificial ventilation. Discharge decision-making and provision of tracheostomy support on the ward was according to local usual practice. Detailed inclusion criteria and other study details are published.[8] There were no missing data. The TracMan trial was registered on the ISRCTN database (ISRCTN28588190).

Patient and Public Involvement

Patient and Public Involvement was not sought for this study using a clinical trial database.

Definitions

We included patients who received a tracheostomy in either arm of the TracMan study. We excluded patients randomised in error or withdrawn from the trial before 30 days. We also excluded patients transferred to another ICU or discharged to another hospital or home. We defined two groups. The "discharged with a tracheostomy" group had left an ICU with a tracheostomy in place. They went to a ward or a high dependency unit (HDU) in the same hospital. The "remained in ICU" group had either been decannulated before they left the ICU or died in the ICU with the tracheostomy in place.

For peer review only - http://bmjopen.bmj.com/site/about/guidelines.xhtml

For peer review only

6

Development of propensity models

We chose propensity matching to produce a "remained in ICU" population as similar as possible to the population "discharged with a tracheostomy". This prioritised matching the populations above the individual matching of patients (as would occur with individual patient matching). We developed two propensity models using logistic regression. One model was for discharge to a general ward with a tracheostomy in place. The other model was for discharge to an HDU with a tracheostomy in place. We chose potential confounders from those available within the TracMan dataset. The original TracMan dataset was chosen by an expert trial team and underwent stringent external review. We undertook a literature review to inform the choices made. The following variables were included in the propensity model (as potential confounders of the relationship between leaving an ICU with a tracheostomy in place and short term mortality):

- Age on admission to the ICU
- APACHE II Acute Physiology Score on ICU admission
- Surgical status (admission from an operating theatre following elective/scheduled surgery; admission from theatre following emergency/urgent surgery; non-surgical)
- Underlying reason for mechanical ventilation (neurological/non-neurological)
- Number of days since tracheostomy (up to and including the day of discharge for cases, or matched day for controls)
- Number of days receiving an intravenous bolus dose or an infusion of drugs primarily for sedation (up to and including the day of discharge for cases, or matched day for matched controls)
- Receiving an intravenous bolus dose or an infusion of drugs primarily for sedation on the day of discharge for cases, or matched day for matched controls
- Number of days of support (up to and including the day of discharge for cases, or matched day for matched controls) of the following organ systems:

For peer review only - http://bmjopen.bmj.com/site/about/guidelines.xhtml

- ○ Advanced respiratory support (invasive artificial ventilation; extracorporeal gas exchange)
- ○ Basic respiratory support (mask continuous positive airway pressure [CPAP]; non-invasive ventilation; >50% inspired oxygen; recent extubation following prolonged mechanical ventilation; intubation to protect the airway; physiotherapy to clear secretions at least two hourly)
- ○ Circulatory support (vasoactive drug infusion; circulatory instability due to hypovolaemia; patients resuscitated following cardiac arrest; intra-aortic balloon pump in place)
- ○ Neurological support (central nervous system depression sufficient to prejudice the airway; invasive neurological monitoring)
- ○ Renal support (acute renal replacement therapy).

- Organ support (as above) received on the day of discharge for cases, or matched day for controls.

Matching

We matched patients discharged with a tracheostomy (cases) 1:1 with the patient (and day) from the "remained in ICU" group (controls). We matched to the patient with the closest propensity score (predicted log odds from the propensity model). We matched patients up to a difference of 1 standard deviation of the propensity score. We used the ward or HDU propensity model according to the patient's discharge destination.

To ensure comparability between the two groups, the "remained in ICU" group were required to: have had the tracheostomy for at least two days by the matched day; have not received advanced respiratory support on the day following the matched day; and (for controls matched to patients discharged to a ward with the tracheostomy) have not received neurological support or renal support on the day following the matched day. We give our definitions of organ support below.

For peer review only - http://bmjopen.bmj.com/site/about/guidelines.xhtml

For peer review only

Outcome

The primary outcome was 30-day mortality from the matching day. For the "discharged with a tracheostomy" group this was death within 30 days after the discharge day. For the "remained in ICU" group, this was death within 30 days after the matched day.

Assessment of balance

We compared cases and controls before and after matching, for their age, sex, APACHE II Acute Physiology Score, overall APACHE II Score, surgical status, underlying reason for mechanical ventilation, number of days since tracheostomy, number of days receiving sedatives, number of days of organ support, and organ support received on matched day. We compared continuous variables with mean and standard deviation. We compared categorical variables with number and percent. We compared balance with the standardised difference. We defined the standardised difference as the absolute value of the difference in means divided by the pooled standard deviation.[9] In addition, we used quantile-quantile (QQ) plots to assess the balance across the full distribution for age, APACHE II score and propensity score. We assessed balance for the full cohort and split by the case's destination following discharge (ward or HDU).

Statistical Analysis

We estimated the relative risk of 30-day mortality for cases compared with matched controls by Poisson regression, conditional on the matched data.[10] We estimated standard errors with the non-parametric bootstrap.[11] We calculated relative risks both unadjusted and adjusted for age, APACHE II Acute Physiology Score and respiratory support on the matched day, to control for any remaining imbalance in these risk factors. We evaluated any difference in effect between patients discharged to a ward and those discharged to an HDU by introducing an interaction term in the Poisson regression model. We performed all analyses using Stata/SE Version 13.0 (StataCorp LP, College Station, Texas, USA).

For peer review only

RESULTS

909 patients were enrolled in the TracMan trial between November 2004 and November 2008. We excluded 10 patients randomised in error or withdrawn from the trial before 30 days. Of the remaining 899 patients, 622 (69.2%) received a tracheostomy. 174 deaths in ICU occurred in this group. 144 patients had a tracheostomy in place when they left an ICU. 70 (11.3%) were discharged to a ward in the same hospital. 74 (11.9%) were discharged to an HDU in the same hospital. We included 999 days of observation from 294 patients in the control pool. For the ward control pool we included 960 days of observation from 292 patients (as we did not include days where patients received neurological or renal support). Figure 1 shows the selection process for cases and controls.

Table 1 shows balance statistics for the full cohort, before and after matching. Balance statistics for those discharged to a ward and those discharged to an HDU are included in the supplementary material – Tables S1 and S2. There was imbalance in many covariates before matching (as indicated by standardised difference values above 10). Balance improved after matching. Figure 2 shows QQ plots for age, APACHE II score and propensity score. The QQ plot of propensity score balance shows almost all patients were closely matched.

Remaining in an ICU for tracheostomy care did not statistically affect mortality in comparison to care outside an ICU (Table 2).

For peer review only

For peer review only - http://bmjopen.bmj.com/site/about/guidelines.xhtml

Table 1 Balance in potential confounders before and after matching – full cohort

	Before matching			After matching		
	Discharged with tracheostomy (N=144)	Remained in ICU (N=999[a])	Standardised difference	Discharged with tracheostomy (N=144)	Remained in ICU (N=144[b])	Standardised difference
Age (years), mean (SD)	64.5 (12.5)	62.7 (13.1)	14.1	64.5 (12.5)	65.4 (11.2)	7.2
Male, n (%)	82 (56.9)	644 (64.5)	15.4	82 (56.9)	104 (72.2)	32.2
APACHE II APS, mean (SD)	14.4 (5.8)	13.4 (6.5)	16.2	14.4 (5.8)	13.7 (6.7)	11.4
APACHE II Score, mean (SD)	19.2 (6.8)	17.9 (7.0)	19.3	19.2 (6.8)	18.8 (7.3)	5.6
Surgical status – elective/scheduled, n (%)	8 (5.6)	51 (5.1)	2.0	8 (5.6)	10 (6.9)	5.7
Surgical status – emergency/urgent, n (%)	24 (16.7)	140 (14.0)	7.4	24 (16.7)	25 (17.4)	1.8
Neurological reason for ventilation, n (%)	18 (12.5)	100 (10.0)	7.9	18 (12.5)	19 (13.2)	2.1
Days since tracheostomy, mean (SD)	14.6 (10.4)	17.2 (13.6)	21.4	14.6 (10.4)	14.1 (10.9)	4.6
Days receiving sedatives, mean (SD)	8.1 (6.9)	9.1 (6.9)	14.6	8.1 (6.9)	7.8 (5.7)	4.6
Days of advanced respiratory support, mean (SD)	15.3 (10.9)	19.0 (14.1)	29.2	15.3 (10.9)	15.1 (11.9)	1.8
Days of basic respiratory support, mean (SD)	5.9 (9.5)	5.5 (9.5)	4.5	5.9 (9.5)	6.8 (11.9)	8.3
Days of circulatory support, mean (SD)	3.4 (3.7)	4.3 (4.7)	21.2	3.4 (3.7)	3.2 (3.2)	5.0
Days of neurological support, mean (SD)	3.5 (6.6)	2.4 (5.3)	18.9	3.5 (6.6)	3.0 (5.2)	10.0
Days of renal support, mean (SD)	1.2 (4.2)	2.3 (6.6)	19.9	1.2 (4.2)	0.9 (3.0)	8.8
Receiving sedatives on final day, n (%)	8 (5.6)	92 (9.2)	14.0	8 (5.6)	7 (4.9)	3.1
Advanced respiratory support on final day, n (%)	29 (20.1)	322 (32.2)	27.7	29 (20.1)	21 (14.6)	14.7
Basic respiratory support on final day, n (%)	89 (61.8)	612 (61.3)	1.1	89 (61.8)	90 (62.5)	1.4
Circulatory support on final day, n (%)	0 (0)	32 (3.2)	25.7	0 (0)	0 (0)	N/A
Neurological support on final day, n (%)	6 (4.2)	22 (2.2)	11.2	6 (4.2)	2 (1.4)	16.9
Renal support on final day, n (%)	0 (0)	37 (3.7)	27.7	0 (0)	0 (0)	N/A

[a]999 days of observation from 294 patients; [b]84 unique patients after matching with replacement

APACHE, Acute Physiology And Chronic Health Evaluation; APS, Acute Physiology Score; SD, standard deviation

For peer review only

Table 2 Outcome of 30-day mortality following discharge with a tracheostomy in place compared with those who remained in an ICU

	30-day mortality, deaths (%) [95% CI]		Unadjusted RR [95% CI]	P value	Adjusted RR[a] [95% CI]	P value
	Discharged with tracheostomy	Remained in ICU				
Overall (N=144)	22 (15.3) [10.3, 22.0]	26 (18.1) [12.6, 25.1]	0.85 [0.49, 1.47]	0.55	0.98 [0.43, 2.23]	0.96
Discharged to a ward (N=70)	13 (18.6) [11.2, 29.2]	11 (15.7) [9.0, 26.0]	1.18 [0.48, 2.89]	0.48[b]	1.46 [0.45, 4.78]	0.37[b]
Discharged to an HDU (N=74)	9 (12.2) [6.5, 21.5]	15 (20.3) [12.7, 30.8]	0.60 [0.11, 3.30]		0.61 [0.15, 2.57]	

[a]Adjusted for age and APACHE II Acute Physiology Score and respiratory support on final day; [b]P value for test of interaction with destination following discharge

CI, confidence interval; RR, relative risk

For peer review only

For peer review only - http://bmjopen.bmj.com/site/about/guidelines.xhtml

DISCUSSION

There is little doubt that hospital mortality after leaving an ICU is high in patients with a new tracheostomy.[1, 2, 7] Our study supports these previous findings. Patients leaving ICU with a tracheostomy had a 30 day mortality nearly twice the 9.5% in-hospital mortality for all patients discharged from UK ICUs at the time.[12] There are two possible explanations for this high mortality. There may be deficiencies in tracheostomy care outside ICU, which staying in an ICU would avoid. Alternatively, having a tracheostomy when leaving an ICU may simply mark a higher risk group. To separate these two possibilities, we propensity score matched patients who left an ICU with a tracheostomy with patients who remained in an ICU with a tracheostomy. The TracMan database contained high quality daily patient information. These data allowed us to match on time and treatment periods as well as static variables. Patients required similar levels of support when matched. We found no significant difference in post-ICU 30-day mortality. This remained true when we separated patients discharged to a general ward and those discharged to an HDU.

Our propensity score matched cohort design limits the study findings. Although balance was improved between groups after matching, imbalances remained. This was always likely given the number of factors matched. Our results may be confounded by an unknown and unrecorded variable that alters both the likelihood of leaving an ICU with a tracheostomy and subsequent death. This is a risk in all similarly designed studies. Our detailed database, allowing us to match on multiple variables, should have minimised this risk. The original TracMan study did not collect detailed information on post-tracheostomy care. However, the large number of sites taking part in the study would suggest that this care represented common UK practice.

The data used come from a clinical trial, in which no deaths were attributed to the tracheostomy procedure. Variables were collected using a comprehensive set of definitions to ensure consistency. Out-of-range or missing data were checked during the trial with source documents. These strategies provided a robust data set for analysis. However, the database does not include all patients who had

For peer review only - http://bmjopen.bmj.com/site/about/guidelines.xhtml

a tracheostomy at the centres during the study period. Some patients will have received a tracheostomy outside the study. Patients in a clinical trial may experience closer monitoring, and therefore different care, than in usual practice.[13] However, the TracMan study did not require ward or High Dependency Unit follow-up by research staff. The risk that researcher staff directly affected ward care appears small.

Even with a 909-patient database available from a 72-centre study of tracheostomy timing we were only able to generate 144 cases, limiting the power of the study to detect an effect of remaining on an ICU for tracheostomy care. The wide confidence intervals around our estimates mean we cannot exclude a clinically relevant effect. Cases were matched 1:1 with controls. Matching at one case to two controls may have increased power slightly. Conversely, as we used replacement this would also have increased the duplication of patients in the control group.

Although these concerns place some limitations on the generalisability of our study, the results remain important. Despite being over 10 years old, the TracMan database is a rich source from which to further our knowledge about patients who receive a tracheostomy whilst on an ICU. We are not aware of a similar prospective database. Our study benefits from the large number (622) of patients who underwent a tracheostomy within the TracMan trial, and the prospective daily information gathered. These factors allowed a large pool of patients from which to match. As a result, almost all patients were closely matched. The scale of the database allowed us to include substantially more patients from many more centres than has previously been possible.

A previous multi-centre study took a different propensity-based approach. They compared 141 patients leaving an ICU with a tracheostomy in place with 60 leaving after tracheostomy removal.[2] No difference in hospital mortality *after leaving* an ICU was found. Our study assesses the effect of tracheostomy care *in an ICU* in comparison to care outside an ICU at an equivalent point in patients' recovery. Our approach has the advantage that mortality whilst on ICU receiving tracheostomy care is taken into account. Neither approach suggests that mortality is increased by tracheostomy care

For peer review only - http://bmjopen.bmj.com/site/about/guidelines.xhtml

For peer review only

outside an ICU. The two propensity-based studies have findings that contrast with previous research.[6, 7] The findings demonstrate the importance of taking imbalances in other risk factors into account.

Two other information sources support the view that the mortality attributable to the tracheostomy and its care after leaving an ICU may be low. A study reviewing 453 incidents related to tracheostomies in patients treated on hospital wards reported to the UK National Patient Safety Agency over two years[14] found only 15 (3%) where the incident was associated with the death of a patient. In a national audit only 5 of 518 (1%) ward patients with a tracheostomy were classified as having suffered harm from tracheostomy-related problems. Only one of these suffered a cardiac arrest.[15]

The question of where to provide care for patients who have had a tracheostomy placed on an ICU might best be answered by a randomised controlled trial. However, the incidence of events in our study and those of others suggest a very large (and so expensive) trial would be required.[2] At present, a propensity-matched approach may provide the highest level of evidence practically available.

Recommendations for improving ward outcomes for patients leaving an ICU with a tracheostomy have focused on tracheostomy care.[15-17] Ensuring appropriate tracheostomy care and training for tracheostomy-related emergencies is clearly important. In undertaking a large multi-centre randomised controlled trial, a limited number of outcomes can be collected. Individual hospital tracheostomy care was not collected in the study. Other problems underlie the continued need for a tracheostomy on the ward. Our findings indicate that remaining in an area of high staff ratio and skill mix, suggesting safe tracheostomy management, did not improve mortality. Therefore, mortality may be more related to the underlying diseases and conditions than the tracheostomy itself. We need better understanding of these issues to improve outcomes for these vulnerable patients.

For peer review only - http://bmjopen.bmj.com/site/about/guidelines.xhtml

CONCLUSION

In a propensity-based analysis of a large prospective dataset, 30-day mortality was high in patients who could be discharged from an ICU with a tracheostomy in place. However, keeping patients on an ICU to provide tracheostomy care did not affect mortality in comparison to ward-based care. Tracheostomy presence may indicate a higher risk of mortality due to underlying diseases and conditions rather than posing a risk in itself. This should be considered when planning care for patients with a tracheostomy.

ACKNOWLEDGEMENTS

The full list of TracMan trial collaborators has been published and their major contribution to this study is acknowledged. The TracMan trial was registered on the ISRCTN database (ISRCTN28588190).

FUNDING

The work described in this paper was funded by the National Institute for Health Research (NIHR) Oxford Biomedical Research Centre (BRC) and by the Intensive Care National Audit and Research Centre. The views expressed are those of the author(s) and not necessarily those of the NHS, the NIHR or the Department of Health. The original TracMan study was funded by the UK Intensive Care Society and the Medical Research Council.

DECLARATION OF COMPETING INTERESTS

Peter Watkinson co-developed the System for Electronic Notification and Documentation (SEND), for which Sensyne Health has purchased a sole licence. The company has a research agreement with the University of Oxford and royalty agreements with Oxford University Hospitals NHS Trust and the University of Oxford. Sensyne Health may in the future pay Peter Watkinson personal fees. Peter Watkinson was Chief Medical Officer for Sensyne Health. No other authors have any COI.

For peer review only

16

CONTRIBUTION STATEMENT

DY, PW and SV conceived the original idea. SV and DY prepared the manuscript with revisions from PW and DH. All authors read and approved the final manuscript. All authors had full access to all of the data (including statistical reports and tables) in the study and take responsibility for the integrity of the data and the accuracy of the data analysis.

DATA SHARING STATEMENT

Deidentified data may be made available on reasonable request to the corresponding author.

For peer review only

REFERENCES

[1] Clec'h C, Alberti C, Vincent F, et al. Tracheostomy does not improve the outcome of patients requiring prolonged mechanical ventilation: a propensity analysis. *Crit Care Med* 2007;35:132-8.

[2] Fernandez R, Tizon AI, Gonzalez J, et al. Intensive care unit discharge to the ward with a tracheostomy cannula as a risk factor for mortality: a prospective, multicenter propensity analysis. *Crit Care Med* 2011;39:2240-5.

[3] Nathens AB, Rivara FP, Mack CD, et al. Variations in rates of tracheostomy in the critically ill trauma patient. *Crit Care Med* 2006;34:2919-24.

[4] Mehta AB, Syeda SN, Bajpayee L, et al. Trends in Tracheostomy for Mechanically Ventilated Patients in the United States, 1993-2012. *AJRCCM* 2015;192:446-54.

[5] Freeman BD, Morris PE. Tracheostomy practice in adults with acute respiratory failure. *Crit Care Med* 2012;40:2890-6.

[6] Araujo I, Goncalves-Pereira J, Teixeira S, et al. Assessment of risk factors for in-hospital mortality after intensive care unit discharge. *Biomarkers* 2012;17:180-5.

[7] Fernandez R, Bacelar N, Hernandez G, et al. Ward mortality in patients discharged from the ICU with tracheostomy may depend on patient's vulnerability. *Intensive Care Med* 2008;34:1878-82.

For peer review only - http://bmjopen.bmj.com/site/about/guidelines.xhtml

[8] Young D, Harrison DA, Cuthbertson BH, et al. Effect of early vs late tracheostomy placement on survival in patients receiving mechanical ventilation: the TracMan randomized trial. *JAMA* 2013;309(20):2121-9.

[9] Austin PC. Balance diagnostics for comparing the distribution of baseline covariates between treatment groups in propensity-score matched samples. *Stat Med* 2009;28(25):3083-107.

[10] Cummings PBM. Analysis of matched cohort data. *Stata J* 2004;4:274-81.

[11] Cummings P, McKnight B, Greenland S. Matched cohort methods for injury research. *Epidemiol Rev* 2003;25:43-50.

[12] ICNARC: Intensive Care National Audit & Rresearch Centre (ICNARC). Case Mix Programme Summary Statistics 2008-09. www.icnarc.org/Our-Audit/Audits/Cmp/Reports/Summary-Statistics. (Acessed 14/02/2020).

[13] McCarney R, Warner J, Iliffe S, et al. The Hawthorne Effect: a randomised, controlled trial. *BMC Med Res Methodol* 2007;7:30.

[14] McGrath BA, Thomas AN. Patient safety incidents associated with tracheostomies occurring in hospital wards: a review of reports to the UK National Patient Safety Agency. *Postgrad Med J* 2010; 86:522-5.

[15] Wilkinson KA, Martin IC, Freeth H, et al. On the Right Trach? A review of the care received by patients who underwent a tracheostomy. A report by the National Confidential Enquiry into Patient Outcome and Death. 2014.

[16] The global tracheostomy collaborative. http://globaltrach.org/. (Accessed 14/02/2020).

[17] National Tracheostomy Safety Project. www.tracheostomy.org.uk. (Accessed 14/02/2020).

Figure legends:

For peer review only - http://bmjopen.bmj.com/site/about/guidelines.xhtml

Figure 1: Case and control selection flow diagram

Figure 2: Quantile-quantile (QQ) plots of balance in (A) age, (B) APACHE II Acute Physiology Score and (C) propensity score

For peer review only

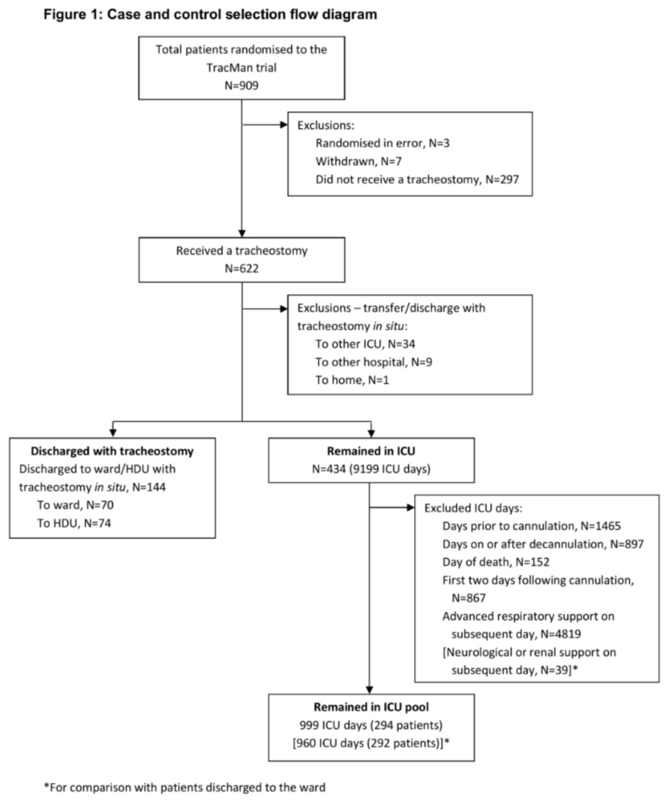

Figure 1: Case and control selection flow diagram

Figure 2 Quantile-quantile (QQ) plots of balance in (A) age, (B) APACHE II Acute Physiology Score and (C) propensity score

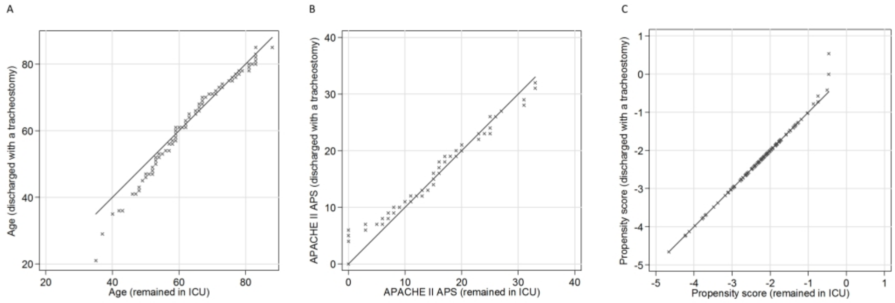

Figure 2: Quantile-quantile (QQ) plots of balance in (A) age, (B) APACHE II Acute Physiology Score and (C) propensity score

SUPPLEMENTARY MATERIAL: Balance in potential confounders

Table S1 Balance in potential confounders before and after matching – cases discharged to a ward

Potential confounder	Before matching			After matching		
	Discharged with tracheostomy (N=70)	Remained in ICU (N=960[a])	Standardised difference	Discharged with tracheostomy (N=70)	Remained in ICU (N=70[b])	Standardised difference
Age (years), mean (SD)	65.0 (13.3)	63.3 (12.9)	13.1	65.0 (13.3)	66.3 (10.3)	10.9
Male, n (%)	50 (71.4)	607 (63.2)	17.5	50 (71.4)	48 (68.6)	6.2
APACHE II APS, mean (SD)	14.7 (6.2)	13.2 (6.5)	25.0	14.7 (6.2)	14.9 (6.7)	3.3
APACHE II Score, mean (SD)	19.5 (7.1)	17.7 (7.0)	25.8	19.5 (7.1)	20.0 (7.4)	6.7
Surgical status – elective/scheduled, n (%)	2 (2.9)	51 (5.3)	12.4	2 (2.9)	2 (2.9)	0
Surgical status – emergency/urgent, n (%)	10 (14.3)	139 (14.5)	0.5	10 (14.3)	10 (14.3)	0
Neurological reason for ventilation, n (%)	11 (15.7)	94 (9.8)	17.7	11 (15.7)	10 (14.3)	4.0
Days since tracheostomy, mean (SD)	15.7 (11.0)	17.2 (13.7)	11.8	15.7 (11.0)	15.5 (11.8)	1.5
Days receiving sedatives, mean (SD)	7.6 (5.1)	8.9 (6.7)	23.1	7.6 (5.1)	7.5 (5.3)	1.6
Days of advanced respiratory support, mean (SD)	14.7 (9.4)	19.1 (14.3)	36.0	14.7 (9.4)	14.6 (12.3)	0.9
Days of basic respiratory support, mean (SD)	6.1 (7.7)	5.6 (9.7)	6.0	6.1 (7.7)	6.3 (11.0)	1.8
Days of circulatory support, mean (SD)	3.2 (3.4)	4.1 (4.6)	23.5	3.2 (3.4)	2.6 (2.7)	20.0
Days of neurological support, mean (SD)	3.7 (6.2)	2.3 (5.1)	26.1	3.7 (6.2)	2.9 (4.3)	16.0
Days of renal support, mean (SD)	0.9 (4.6)	1.6 (5.4)	14.1	0.9 (4.6)	0.7 (2.6)	7.3
Receiving sedatives on final day, n (%)	6 (8.6)	83 (8.6)	0.3	6 (8.6)	4 (5.7)	11.0
Advanced respiratory support on final day, n (%)	4 (5.7)	310 (32.3)	71.9	4 (5.7)	5 (7.1)	5.8
Basic respiratory support on final day, n (%)	46 (65.7)	593 (61.8)	8.2	46 (65.7)	40 (57.1)	17.6
Circulatory support on final day, n (%)	0 (0)	32 (3.3)	26.2	0 (0)	0 (0)	N/A
Neurological support on final day, n (%)	6 (8.6)	11 (1.1)	34.8	6 (8.6)	2 (2.9)	24.6
Renal support on final day, n (%)	0 (0)	8 (0.8)	13.0	0 (0)	0 (0)	N/A

For peer review only

[a]960 days of observation from 292 patients; [b]49 unique patients after matching with replacement

APACHE, Acute Physiology And Chronic Health Evaluation; APS, Acute Physiology Score; SD, standard deviation

Table S2 Balance in potential confounders before and after matching – cases discharged to an HDU

Potential confounder	Before matching			After matching		
	Discharged with tracheostomy (N=74)	Remained in ICU (N=999[a])	Standardised difference	Discharged with tracheostomy (N=74)	Remained in ICU (N=74[b])	Standardised difference
Age (years), mean (SD)	64.1 (11.8)	62.7 (13.1)	11.0	64.1 (11.8)	64.5 (11.9)	3.5
Male, n (%)	32 (43.2)	644 (64.5)	43.4	32 (43.2)	56 (75.7)	69.5
APACHE II APS, mean (SD)	14.1 (5.5)	13.4 (6.5)	11.5	14.1 (5.5)	12.5 (6.5)	26.5
APACHE II Score, mean (SD)	18.9 (6.5)	17.9 (7.0)	15.5	18.9 (6.5)	17.7 (7.0)	18.3
Surgical status – elective/scheduled, n (%)	6 (8.1)	51 (5.1)	12.1	6 (8.1)	8 (10.8)	9.2
Surgical status – emergency/urgent, n (%)	14 (18.9)	140 (14.0)	13.2	14 (18.9)	15 (20.3)	3.4
Neurological reason for ventilation, n (%)	7 (9.5)	100 (10.0)	1.9	7 (9.5)	9 (12.2)	8.7
Days since tracheostomy, mean (SD)	13.6 (9.9)	17.2 (13.6)	30.5	13.6 (9.9)	12.8 (9.8)	7.9
Days receiving sedatives, mean (SD)	8.7 (8.2)	9.1 (6.9)	6.1	8.7 (8.2)	8.2 (6.0)	6.8
Days of advanced respiratory support, mean (SD)	15.9 (12.2)	19.0 (14.1)	23.5	15.9 (12.2)	15.6 (11.5)	2.5
Days of basic respiratory support, mean (SD)	5.8 (11.0)	5.5 (9.5)	2.7	5.8 (11.0)	7.4 (12..8)	13.2
Days of circulatory support, mean (SD)	3.5 (4.1)	4.3 (4.7)	16.7	3.5 (4.1)	3.8 (3.5)	6.4
Days of neurological support, mean (SD)	3.4 (7.0)	2.4 (5.3)	15.3	3.4 (7.0)	3.0 (6.0)	5.4
Days of renal support, mean (SD)	1.4 (3.9)	2.3 (6.6)	16.2	1.4 (3.9)	1.0 (3.3)	10.2
Receiving sedatives on final day, n (%)	2 (2.7)	92 (9.2)	27.7	2 (2.7)	3 (4.1)	7.4
Advanced respiratory support on final day, n (%)	25 (33.8)	322 (32.2)	3.3	25 (33.8)	16 (21.6)	27.2
Basic respiratory support on final day, n (%)	43 (58.1)	612 (61.3)	6.4	43 (58.1)	50 (67.6)	19.5
Circulatory support on final day, n (%)	0 (0)	32 (3.2)	25.7	0 (0)	0 (0)	N/A
Neurological support on final day, n (%)	0 (0)	22 (2.2)	21.2	0 (0)	0 (0)	N/A

For peer review only

Renal support on final day, n (%)	0 (0)	37 (3.7)	27.7	0 (0)	0 (0)	N/A

[a]999 days of observation from 294 patients; [b]52 unique patients after matching with replacement

APACHE, Acute Physiology And Chronic Health Evaluation; APS, Acute Physiology Score; SD, standard deviation

For peer review only

STROBE Statement—checklist of items that should be included in reports of observational studies

	Item No	Recommendation	Page
**Title and abstract**	1	(*a*) Indicate the study's design with a commonly used term in the title or the abstract	1
		(*b*) Provide in the abstract an informative and balanced summary of what was done and what was found	2
**Introduction**			
Background/rationale	2	Explain the scientific background and rationale for the investigation being reported	4
Objectives	3	State specific objectives, including any prespecified hypotheses	4
**Methods**			
Study design	4	Present key elements of study design early in the paper	5-6
Setting	5	Describe the setting, locations, and relevant dates, including periods of recruitment, exposure, follow-up, and data collection	5
Participants	6	(*a*) *Cohort study*—Give the eligibility criteria, and the sources and methods of selection of participants. Describe methods of follow-up<br>*Case-control study*—Give the eligibility criteria, and the sources and methods of case ascertainment and control selection. Give the rationale for the choice of cases and controls<br>*Cross-sectional study*—Give the eligibility criteria, and the sources and methods of selection of participants	5 – basic, detailed in other paper
		(*b*) *Cohort study*—For matched studies, give matching criteria and number of exposed and unexposed<br>*Case-control study*—For matched studies, give matching criteria and the number of controls per case	6-7
Variables	7	Clearly define all outcomes, exposures, predictors, potential confounders, and effect modifiers. Give diagnostic criteria, if applicable	4-7
Data sources/ measurement	8*	For each variable of interest, give sources of data and details of methods of assessment (measurement). Describe comparability of assessment methods if there is more than one group	5
Bias	9	Describe any efforts to address potential sources of bias	8
Study size	10	Explain how the study size was arrived at	n/a – whole cohort of previous study
Quantitative variables	11	Explain how quantitative variables were handled in the analyses. If applicable, describe which groupings were chosen and why	8
Statistical methods	12	(*a*) Describe all statistical methods, including those used to control for confounding	8
		(*b*) Describe any methods used to examine subgroups and interactions	8
		(*c*) Explain how missing data were addressed	4
		(*d*) *Cohort study*—If applicable, explain how loss to follow-up was addressed<br>*Case-control study*—If applicable, explain how matching of cases and controls was addressed<br>*Cross-sectional study*—If applicable, describe analytical methods taking account of sampling strategy	n/a
		(*e*) Describe any sensitivity analyses	8

Continued on next page

For peer review only

**Results**			
Participants	13*	(a) Report numbers of individuals at each stage of study—eg numbers potentially eligible, examined for eligibility, confirmed eligible, included in the study, completing follow-up, and analysed	8-9 and figure 1
		(b) Give reasons for non-participation at each stage	8-9 and figure 1
		(c) Consider use of a flow diagram	Figure 1
Descriptive data	14*	(a) Give characteristics of study participants (eg demographic, clinical, social) and information on exposures and potential confounders	Table 1
		(b) Indicate number of participants with missing data for each variable of interest	n/a
		(c) *Cohort study*—Summarise follow-up time (eg, average and total amount)	n/a
Outcome data	15*	*Cohort study*—Report numbers of outcome events or summary measures over time	
		Case-control study—Report numbers in each exposure category, or summary measures of exposure	Table 1
		Cross-sectional study—Report numbers of outcome events or summary measures	
Main results	16	(*a*) Give unadjusted estimates and, if applicable, confounder-adjusted estimates and their precision (eg, 95% confidence interval). Make clear which confounders were adjusted for and why they were included	Table 2
		(*b*) Report category boundaries when continuous variables were categorized	n/a
		(*c*) If relevant, consider translating estimates of relative risk into absolute risk for a meaningful time period	n/a
Other analyses	17	Report other analyses done—eg analyses of subgroups and interactions, and sensitivity analyses	Supp Tables 1 and 2 HDU/ward
**Discussion**			
Key results	18	Summarise key results with reference to study objectives	12
Limitations	19	Discuss limitations of the study, taking into account sources of potential bias or imprecision. Discuss both direction and magnitude of any potential bias	12-13
Interpretation	20	Give a cautious overall interpretation of results considering objectives, limitations, multiplicity of analyses, results from similar studies, and other relevant evidence	12
Generalisability	21	Discuss the generalisability (external validity) of the study results	13
**Other information**			
Funding	22	Give the source of funding and the role of the funders for the present study and, if applicable, for the original study on which the present article is based	15

For peer review only - http://bmjopen.bmj.com/site/about/guidelines.xhtml
